# Dental and Periodontal Care at the Bedside Using a Portable Dental Unit in Hospitalized Special Needs Patients: The Experience of an Italian Pediatric Hospital

**DOI:** 10.3390/ijerph18157987

**Published:** 2021-07-28

**Authors:** Angela Galeotti, Massimiliano Ciribè, Giorgio Matarazzo, Giancarlo Antonielli, Paola Festa, Alessandro Inserra, Annelyse Garret-Bernardin, Michele Callea, Massimiliano Raponi

**Affiliations:** 1 Unit of Dentistry, Bambino Gesù Children’s Research Hospital IRCCS, 00165 Rome, Italy; angela.galeotti@opbg.net (A.G.); massimiliano.ciribe@opbg.net (M.C.); giorgio.matarazzo@opbg.net (G.M.); giancarlo.antonielli@opbg.net (G.A.); paolafesta1@gmail.com (P.F.); annelyse.garret@opbg.net (A.G.-B.); 2 Department of General and Thoracic Surgery, Bambino Gesù Children’s Research Hospital IRCCS, 00165 Rome, Italy; alessandro.inserra@opbg.net; 3 Medical Direction, Bambino Gesù Children’s Hospital, IRCCS, 00165 Rome, Italy; massimiliano.raponi@opbg.net

**Keywords:** portable dental unit, dentistry, special needs patients

## Abstract

Patients with special needs (SNPs) include individuals who are disabled due to physical limitations, medical complications, developmental problems, and cognitive impairments. SNPs may be at an increased risk of oral diseases throughout their lifetime. These patients have difficulties in accessing traditional dental studios or clinics. Moreover, orodental problems may cause local and generalized infections, leading to worrisome complications when not properly treated. In this paper, we describe the preliminary experience of treating dental problems in a series of nine hospitalized patients with special needs. This innovative protocol at the Bambino Gesù Children’s Hospital (Rome, Italy) provides an introduction to a portable dental unit in order to perform oral care for hospitalized patients at the bedside. A multidisciplinary team composed of pediatric dentists, dental hygienists, nursing staff, and the patient’s case manager was involved in the operative protocol. The SNPs described were affected by congenital heart or oncohematological diseases and neurodisabilities, and they were all hospitalized for different reasons: Open heart surgery, chemotherapy, organ transplantation, and rehabilitation. The oral evaluation was mandatory for ruling out or treating problems that could cause complications. Dental extractions, caries and fracture fillings, sealing, and oral hygiene procedures were performed at the bedside of the patients in the reference unit of their pediatric hospital. The results of this protocol confirm the feasibility of dental procedures at patients’ bedside with portable dental units, encourage implementation of their use, and may represent an actionable model for oral care management in hospitalized SNPs.

## 1. Introduction

Oral cavity health is an important requirement for well-being, as well as for social and esthetic reasons [1]. It becomes indispensable for pediatric patients with special needs (SNPs) [2,3], who are disabled due to suffer from physical limitations, medical complications, developmental problems, and cognitive impairments that heavily limit their autonomy and capacity to attend to daily activities, as well as for critically ill or medically compromised patients who are candidates for oncohematological treatments, cardio-surgery, organ transplantation, or long-term rehabilitation.

Dental problems in such pediatric patients are quite common due to the difficulties in maintaining oral hygiene. Moreover they may represent important comorbidities that have an adverse impact on the clinical outcome [4]. Due to the complexity of the basic pathology, SNPs are rarely treated in private dental settings and usually require personalized treatments at pediatric hospitals with specialized dentistry units, all medical and surgical specialties, and multidisciplinary teams, including anesthesiology.

Hospitalized patients with special needs (SNPs) are often immobilized and cannot be transported or are at risk of bleeding, meaning that dental care may not be performed in a dental clinic and should to be provided at the bedside with portable dental units [5,6].

Such equipment was first introduced for primary healthcare in developing countries [7], underserved migrant children [8], rural areas [9], hospices and residences for elderly persons [10], and forcedly isolated persons with socioeconomic or geographic barriers [5]. Bambino Gesù Children’s Hospital is one of the major reference pediatric hospitals in Italy for rare and complex diseases, and the Dentistry Unit within the Department of Specialist Surgery has specialized in the dental care of children affected by complex management pathologies for many years.

In this paper, we describe our experience in treating dental problems in patients with special needs by introducing a portable dental unit in order to provide dental care to hospitalized patients at their bedside.

## 2. Material and Methods

**Figure 1:**
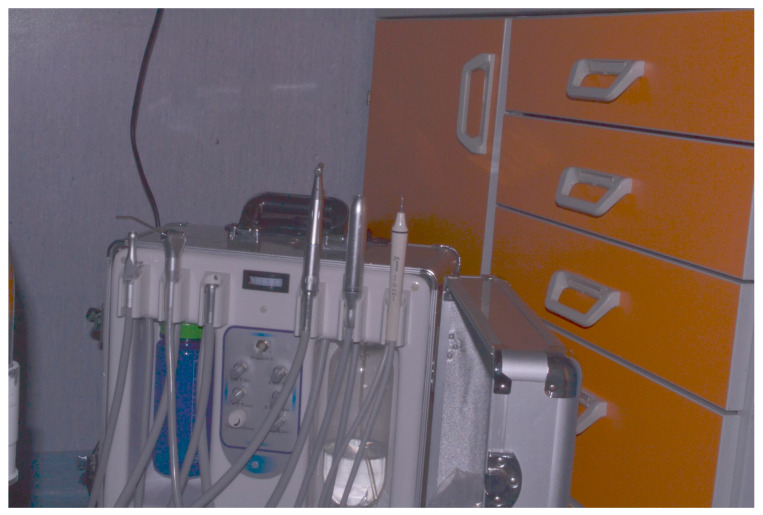
Portable dental unit (Best Dental Unit Ltd. 406).

Dental treatments were carried out at the bedside of nine SNPs using a portable dental unit (Best Dental Unit Ltd. 406, Guanzhou, China) equipped with ancillary devices (Figure 1).

The operating team was composed of pediatric dentists with experience in special dental care, dental hygienists, nursing staff for the special dental service, and the patient’s case manager of the reference unit. The treatments were performed with or without local anesthesia on the basis of individual clinical needs. All treatments were performed on conscious patients. In patients with a functional laryngeal reflex, the patient was placed in the orthopneic position to favor the flow of liquids; instead, in the case of patients with no laryngeal reflex, a small amount of water was used and a small piece of cloth was retained in place by an assistant, behind the tooth treated to reduce the risk of inhalation. In any case, every time bedside treatment was planned, we alerted the colleagues of the reanimation unit, so that they were ready in case of an emergency.

The operators used personal protective equipment for each procedure. The type and timing of dental the procedures were previously agreed with the staff from the hospital reference unit where the patient was hospitalized. Informed consent was always obtained from the patients and/or their caregivers.

In addition to the procedures summarized in Table 1, all patients and parents received instructions on oral hygiene using a toothbrush and mouthwash with a tell–show–do demonstration by dental hygienists in order to ensure adequate daily oral hygiene maneuvers.

The protocol in use for the portable dental unit was approved by the Medical Direction of the Hospital. The cost/benefit evaluation and monitoring has been carried out by the Management and Control Service.

### Enlisted Patients

Eligible patients for this kind of treatment were those patients that could not safely reach the dental cabinet, but needed immediate dental treatment in order to obtain major surgical treatment or avoid further severe infections.

## 3. Case Series Presentation

All of the main clinical data, dental pathology, and treatments performed at the beside of the nine SNPs are summarized in Table 1.

### 3.1. Patient No. 1: 5-Year-Old Patient Affected by Alagille Syndrome

Alagille syndrome is a genetic disorder affecting the liver and heart. Usually, patients present cholestasis and pulmonary vein stenosis [11]. A five-year-old patient presented with severe pulmonary stenosis that did not allow him to leave his bed for prolonged periods of time; moreover, he was affected by long-standing intrahepatic cholestasis and intractable pruritus. The patient was on a waiting list for liver transplantation. Five days before the transplant, according to the protocol, the child underwent a dental consultation that revealed class I decays on 5.5, 6.5, 7.5, and 8.5. In order to investigate the possible presence of infectious focal lesions in the bone context, a panoramic X-ray was requested and carried out with certain difficulty, due to the patient’s respiratory impairment. The X-ray exam confirmed the presence of the aforementioned decays, but did not show any other lesions. Due to the respiratory difficulties, bedside dental treatment was planned with the intention to avoid systemic bacteremia. After administration of local anesthesia, all decays were successfully treated with restorative therapy. After dental treatment, the patient underwent a major surgical procedure for liver transplantation. Subsequent dental examinations showed good level of oral hygiene.

### 3.2. Patient No. 2: 14-Year-Old Patient with Bone Marrow Aplasia

Bone marrow aplasia (BMA) is a severe clinical condition leading to a lack of production of any kind of blood cells [12,13]. This condition could be caused by several pathologies such as leukemia, poisoning, and genetic disorders. Patients with BMA are very fragile and easily subject to infectious diseases. Usually, BMA treatment is represented by bone marrow transplantation. The patient of this clinical report was hospitalized in the Oncohematological Unit for bone marrow transplantation. On oral examination, the child presented with 16 carious lesions: Destructive decays of 3.6 and 4.6, class I decay of 1.6 and 2.6; class II decay of 1.4, 1.5, 2.4, 2.5, 3.4, 3.5, 4.4, and 4.5; and class III decay of 1.3, 2.3, 3.3, and 4.3. An X-ray exam was requested, but the severe risk of infection did not allow the patient to leave his room and reach the X-ray cabinet, nor, of course, the dental cabinet. Thus, bedside treatment was planned in multiple steps: First, an antibiotic therapy with 1 g intramuscular (I.M.) of ceftriaxone was administered; subsequently, 3.6 and 4.6 were extracted at the same time after platelet transfusion to prevent bleeding. After one week of antibiotic therapy, restorative treatment of the other lesions was carried out to prevent the risk of sepsis or the worsening of the dental decay. The procedure was uneventful and the bone marrow transplantation was successful.

### 3.3. Patient No. 3: 17-Year-Old Patient with Shone Complex

Shone complex, or Shone syndrome, is characterized by the simultaneous presence of supravalvular mitral membrane, parachute mitral valve, muscular or membranous subvalvular aortic stenosis, and coarctation of aorta [14]. This peculiar syndrome leads to a progressive lack of hemodynamic stability; in fact, a simple disequilibrium of the pressure gradient, as commonly observable in a normal subject from clinostatism to orthostatism, could represent a serious condition capable of inducing a systemic loss of perfusion. Therapy for this syndrome is based on medical treatment as a bridge for heart transplantation. This 17-year-old patient was on the waiting list for a heart transplant. As protocol requires, an oral examination was performed before the transplant and, during this event, class I decays were found on 1.6, 1.7, 4.6, and 3.6. Unfortunately, it was not possible to obtain an orthopantomography, since the patient was not able to leave his bed. Bedside restorative treatment was planned and, after administration of antibiotic therapy with 1g B.I.D of amoxicillin/clavulanate, under local anesthesia, all of the decays were restored with a calcium hydroxide layer and a light-cured composite. After reconstructive treatment, complete oral hygiene was performed. The patient successfully underwent heart transplantation.

### 3.4. Patient No. 4: 9-Year-Old Patient with Spinal Muscular Atrophy

The term spinal muscular atrophy (SMA) refers to a group of genetic disorders all characterized by degeneration of anterior horn cells and resultant muscle atrophy and weakness. The most common SMA, accounting for over 95% of cases, is an autosomal recessive disorder that results from a homozygous deletion or mutation in the 5q13 chromosome [15]. This 9-year-old patient also suffered from ulcerative colitis, normotensive hydrocephalus, and palate malformation, and he was also PEG-fed and presented a tracheostomization. A dental consultation requested by the Pneumology Unit revealed gingival hypertrophy and multiple deciduous elements apparently close to spontaneous exfoliation. The teeth were retained by the hypertrophic gingivae, representing a continuous inflammatory stimulus for periodontic tissue and a continuous risk of inhalation. Due to the severe systemic disorder, the patient could not be moved from his bed, so bedside treatment was planned, and after local anesthesia administration, eight deciduous teeth were easily removed at the bedside.

### 3.5. Patient No. 5: 14-Year-Old Patient with Nemaline Myopathy

**Figure 2:**
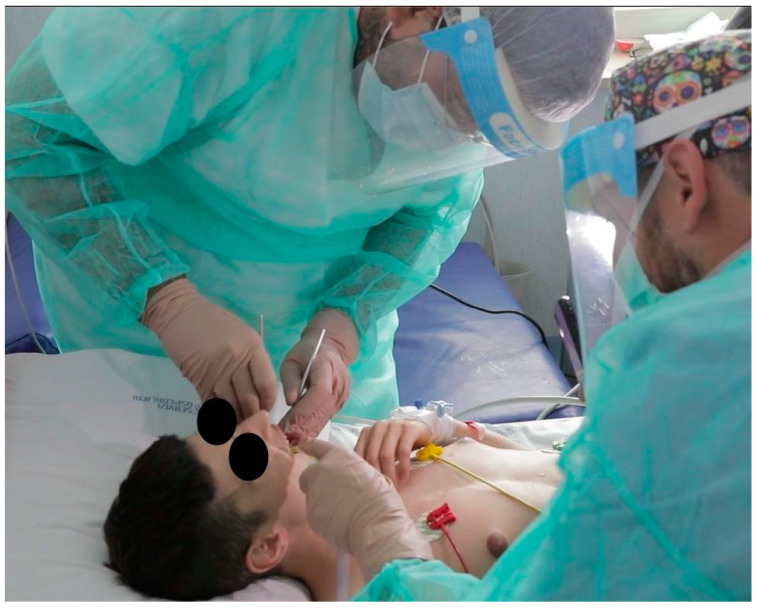
A consultation at the bedside.

**Figure 3:**
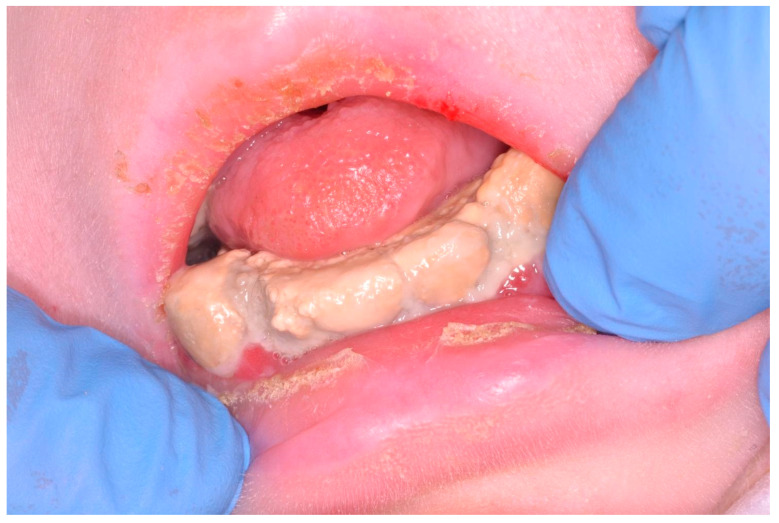
Calculus accumulation and periodontitis in a patient with special needs.

**Figure 4:**
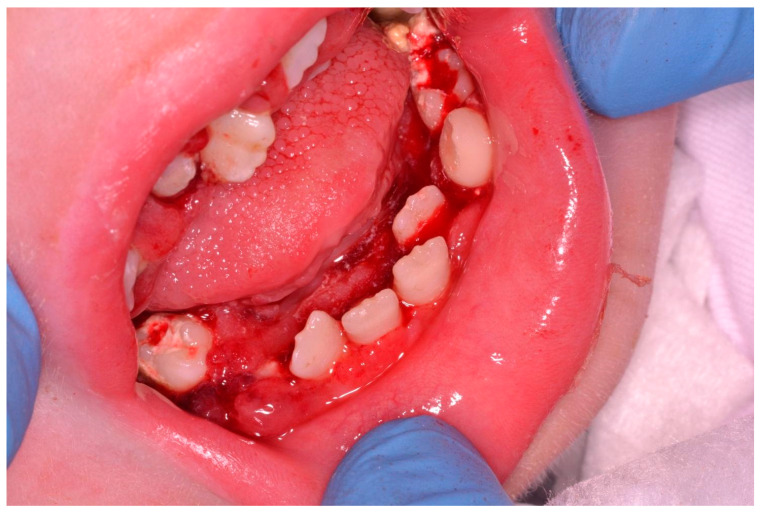
Patient reported in Figure 3 after dental treatment at the bedside.

Nemaline myopathies are a heterogenous group of congenital myopathies caused by de novo, dominantly or recessively inherited, mutations in at least 12 genes. Most patients have congenital onset characterized by muscle weakness and hypotonia, but the spectrum of clinical phenotypes is broad, ranging from severe neonatal presentations to the onset of a milder disorder in childhood. [16] A 14 year-old patient with a nemaline myopathy genetically diagnosed in 2018 had manifested hypotonia since the first months of life. In 2006 and 2007, she underwent positioning of PEG and tracheostomy in another hospital. The child was not able to move, to maintain spontaneous breathing, or to keep her mouth open. Dental examination was requested by colleagues of the Department of Pneumology. Plaque and tartar was found all over the oral cavity, involving all teeth and leading to continuous gingival bleeding. Due to the obvious impossibility of the patient leaving her bed, tartar ablation was planned and carried out uneventfully in serial sessions at the bedside in order to avoid any kind of pulmonary disease potentially derived from the bacterial oropharyngeal population (Figure 2, Figure 3 and Figure 4).

### 3.6. Patient No. 6: 21-Year-Old Patient with Spastic Tetraparesis

Spasticity is a neurological disorder that leads to involuntary contraction of certain muscular groups. A 21-year-old patient with spastic tetraparesis due to perinatal hypoxia was hospitalized at the age of 18 for ab ingestis pneumonia and malnutrition at the Bambino Gesù Multidisciplinary Pediatric Department, where PEG positioning, a fundoplication according to Nissen-Rossetti, and tracheostomy were performed. Three years later, he was hospitalized in the Intensive Care Unit for respiratory failure. In this condition, it was possible to perform an accurate oral examination which previously had not been possible due to noticeable dysphagia and an accentuated vomiting reflex. During the examination, the patient presented calculus stones of sublingual salivary gland and the presence of plaque and tartar. The presence of poor oral hygiene of the patient constituted a risk for the onset of pneumonia derived by oropharyngeal bacteria. He was successfully treated at the bedside with oral hygiene and removal of calculus stones under sedation. This environment represented the safest place for the patient to perform the treatment with absence of reflexes and airway control.

### 3.7. Patient No. 7: 17-Year-Old Patient with Ventricular Arrhythmia

Acute management of patients with ventricular arrhythmia (VA) is aimed at immediate VA termination if the patient is hemodynamically unstable. Prolonged episodes of VA may lead to hemodynamic and metabolic decompensation. Termination is best performed by electrical cardioversion, anti-tachycardia pacing, or defibrillation [17]. A 17-year-old patient was discovered to have cardiac problems during a dental consultation in a GP office. The patient was immediately sent to Bambino Gesù Hospital for cardiologic testing. A paroxystic ventricular arrythmia and a hemopericardium were discovered. The patient also had dental problems: 5.5, 5.4, 6.3, and 7.5 persistence and 1.6 class II decay. The cardiologists decided to proceed with an application of an implantable cardioverter defibrillator (ICD) in order to prevent further episodes of tachyarrhythmia. To proceed with the surgical implantation, dental treatment was mandatory. The patient was hemodynamically unstable and under treatment with anticoagulative and antiarrhythmic drugs, so it was very hazardous to move the patient from his bed. From this perspective, bedside treatment was planned and 5.4, 6.3, and 7.5 were simultaneously pulled at the bedside under local anesthesia. Hemostasis was guaranteed with sutures, hemostatic cloth, and diathermy cauterization. Of course, antibiotic drugs were administrated. After five days, the 1.6 class II decay was restored under local anesthesia at the bedside. After dental treatment, the patient underwent a surgical implantation of an ICD. The patient is in good health and is being followed by cardiologists and oral hygienists.

### 3.8. Patient No. 8: 12-Year-Old Patient with Sickle Cell Disease

Sickle cell disease (SCD) is characterized by intermittent vaso-occlusive events and chronic hemolytic anemia. Vaso-occlusive events result in tissue ischemia, leading to acute and chronic pain, as well as organ damage, that can affect any organ system, including the bones, spleen, liver, brain, lungs, kidneys, and joints [18]. A 12-year-old patient affected by sickle cell disease was admitted to the Immunology Department because of a sickle pain episode related to dental disease. A dental evaluation revealed destructive decay on 8.3 and 5.5 and class I decay on 3.4 and 1.6. After X-ray orthopantomography, the extraction of 8.3 and 5.5 was planned, together with 3.4 and 1.6 restorative treatment. The patient had severe pain in the upper and lower jaws and was undergoing treatment with several drugs via a continuous infusion pump, so he could not leave his bed. Conservative and extractive treatment were carried out on the same day at the bedside under local anesthesia. The pain resolved in a few days with no other drug administration, and the patient was dismissed.

### 3.9. Patient No. 9: 12-Year-Old Patient with Tuberculosis

Tuberculosis is an extremely infectious disease caused by mycobacterium tuberculosis, identified by Robert Koch in 1884. This pathology mainly affects the lungs and requires a prolonged and complex antibiotic treatment. It is also mandatory to place the patient in strict isolation in order to avoid spreading of the infectious disease. A 12-year-old patient was admitted to the Infectious Disease Department with a diagnosis of tuberculosis. She was complaining a severe jaw pain. A dental consultation was requested, and X-ray orthopantomography revealed the presence of class II decay on 3.7 and destructive decay on 3.6. Since the patient was in the active phase of the pathology, it was not possible for her to leave her isolation room. Treatment was planned at the bedside in two steps: The extraction of 3.6 was performed after administration of local anesthesia, and restorative treatment of 3.7 was carried out three days later. After one month, at the end of the antibiotic treatment, the patient was re-evaluated and dismissed.

## 4. Discussion

Children with special needs are predisposed to develop dental problems, especially caries and paradontopathies, because of physical disabilities, excess use of medications, forced oral respiration, and malocclusion that make them unable to maintain adequate oral hygiene [2,3]. Orodental problems that are not properly treated may cause local and generalized infections, leading to worrisome complications [2].

Parallel to the progress of medicine, the life expectancy of children with disabilities, as well as their demand for a odontostomatologic cure, has greatly increased [1,4]. SNPs lack adequate responses to their needs in private practice settings, and most of the time, they require personalized treatment in pediatric hospitals, which are endowed with specialized dentistry units and multidisciplinary teams, including anesthesiology [3,11,12].

During the natural history of a disease, critically ill or medically compromised patients may not be able to leave the bed, so dental consultations and treatments have to be provided at the bedside. Hence, the necessity of employing portable dental units has arisen [6,7].

Until now, the use of portable dental equipment has been reported in elderly patients living in hospices or residential houses [10], in underserved populations in developing countries [7], in migrant children [8], and for the delivery of primary healthcare in rural areas even by adopting indigenously fabricated portable units [9].

This paper described dental treatments performed at the bedside in nine hospitalized patients suffering from different complex diseases. Evaluation of the oral condition is part of the routine management of special needs patients. It has become an indispensable preliminary requirement for oncohematological patients expecting bone marrow (BM) or hematopoietic stem cell (HSC) transplantation, for patients with end-stage organ diseases equally requiring transplantation, as well as for genetic and neurodegenerative diseases [3,13].

For this reason, we upgraded the performance of the Dentistry Unit by activating a portable dental unit to treat bedside-immobilized or not transportable patients in their hospital reference unit. Sometimes, treatment of these patients may not be performed under general anesthesia, because it would expose the patient to life-threatening risk. One of the strengths of the described protocol consists of the treatment of patients within their hospital context, which guarantees greater protection in the case of complications. For this reason, we do not recommend the use of portable dental units at home in patients suffering from complex pathologies.

## 5. Conclusions

In this paper, we reported a pilot study and the preliminary results obtained from nine hospitalized patients with special needs, where different dental procedures were carried out: Teeth extractions, filling of caries or dental fractures, removal of calculus stones, hygiene, and educational healthcare.

The advantages of bedside treatment were a rapid dental care access to highly medically complex pathologies, thus avoiding postponement of the treatment of painful or esthetic conditions and decreasing the use of operatory rooms. Moreover, positive feedback was recorded in terms of comfort and compliance from the patients or their parents, a gain in trust between operators from the various involved units, reinforcement of interdepartmental interaction, and an increase in dentists’ self- confidence toward affordable challenges. These strengths minimize the few apparently weak points that reflect the usual criticality of any innovation, such as logistics, transport of material, staff mobility, and remote reporting.

Nevertheless, portable units suffer some limitations: Their employment should not be prolonged due to overheating of the internal compressor system, and the aspiration system is insufficient, so we always used the aspiration circuit present in the patient’s room.

The results from this case series confirm the feasibility of dental procedures at patients’ bedside with portable dental units, encourage their implementation, and represent an actionable model for other pediatric hospitals.

## Figures and Tables

**Table 1:** Main clinical data, dental pathology, and dental treatment performed at beside in nine SNPs.

Patient No.	1	2	3	4	5	6	7	8	9
Age (years)	5	14	17	9	14	21	17	12	12
Sex	Male	Male	Male	Male	Female	Male	Male	Male	Female
Pathology	Alagille syndrome	Bone marrow aplasia	Shone syndrome	Spinal muscular atrophy	Nemaline myopathy	Spastic tetraparesis	Ventricular arrhythmia	Sickle cell disease	Tuberculosis
Oral pathology	Decay	Decay	Decay	Gingival hypertrophy	Decay	Gingival hypertrophy	Decay	Decay	Decay
X-ray performed	Yes	No	No	No	Yes	No	No	Yes	Yes
Local anesthesia	Yes	Yes	Yes	No	Yes	No	Yes	Yes	Yes
Dental treatment	Restorative treatment	Restorative extractive treatment	Restorative treatment	Oral hygiene	Restorative treatment	Oral hygiene	Restorative extractive treatment	Restorative extractive treatment	Restorative extractive treatment
Full-mouth debridement	No	Yes	Yes	Yes	Yes	Yes	Yes	Yes	Yes

## Data Availability

The datasets used and/or analyzed during the current study are available from the author, Angela Galeotti, on reasonable request.

## References

[1] Ozar D.T. (2006). Basic oral health needs: A public priority. J. Dent. Educ..

[2] Council A.O. (2012). Guidelines on management of dental patients with special health care needs. Pediatr. Dent..

[3] Pini D.D.M., Fröhlich P.C.G.R., Rigo L. (2016). Oral health evaluation in special needs individuals. Einstein (Sao Paulo Braz.).

[4] Chi D.L. (2018). Oral health for US children with special health care needs. Pediatr. Clin..

[5] Charlton D.G., Ehrlich A.D., Minuiotis N.J. (2007). Current update on portable dental equipment. Compend. Contin. Educ. Dent..

[6] Vasjishitha V., Kote S., Basavaraj P., Singla A., Pandita V., Rayneet K.M. (2014). Reach the unreached: A systematic review on mobile dental unit. J. Clin. Diagn. Res..

[7] Ganavadiva R., Chandrashekar B.R., Goel P., Onqual S.G., Jain M. (2014). Mobile and portable dental service catering to the basic oral health needs of the unde3rserved population in developing countries: A proposed model. Ann. Med. Health Sci. Res..

[8] Mulligan R., Seiravan H., Faust F., Habibian M. (2010). Mobile dental clinic: An oral health care delivery model for underserved migrant children. J. Calif. Dent. Assoc..

[9] Goel P., Goel A., Torwane N.A. (2014). Cost-efficiency of indigenously fabricated mobile-portable dental unit in delivery of primary health care in rural India. J. Clin. Diagn. Res..

[10] Lee E.E., Thomas A., Vu T. (2001). Mobile and portable dentistry: Alternative treatment services for the elderly. Spec. Care Dent..

[11] Mitchell E., Gilbert M., Loomes K.M. (2018). Alagille Syndrome. Clin. Liver Dis..

[12] Bromberg M.H., Law E.F., Palermo T.M. (2017). Suicidal Ideation in Adolescents with and without Chronic Pain. Clin. J. Pain.

[13] Wang L., Liu H. (2019). Pathogenesis of aplastic anemia. Hematology.

[14] Elmahrouk A.F., Ismail M.F., Arafat A.A., Dohain A.M., Helal A.M., Hamouda T.E., Galal M., Edrees A.M., Al-Radi O.O., Jamjoom A.A. (2021). Outcomes of biventricular repair for shone’s complex. J. Card. Surg..

[15] Kolb S.J., Kissel J.T. (2015). Spinal Muscular Atrophy. Neurol Clin..

[16] Sewry C.A., Laitila J.M., Wallgren-Pettersson C. (2019). Nemaline myopathies: A current view. J. Muscle Res. Cell Motil..

[17] Deneke T., Nentwich K., Ene E., Berkovitz A., Sonne K., Halbfaß P. (2020). Acute management of ventricular tachycardia. Herzschrittmachertherapie + Elektrophysiologie.

[18] Bender M.A., Adam M.P., Ardinger H.H., Pagon R.A., Wallace S.E., Bean L.J.H., Stephens K., Amemiya A. (2003). Sickle Cell Disease. GeneReviews.

